# X-linked dominant chondrodysplasia punctata due to novel mutation in EBP gene: A case report

**DOI:** 10.1097/MD.0000000000042086

**Published:** 2025-04-04

**Authors:** Xiaoping Shi, Yanxing Lv, Yongjiang Jiang, Pianpian Pan, Yueju Cai

**Affiliations:** a Department of Neonatology, Guangzhou Wowen and Children’s Medical Center, Liuzhou Hospital, Liuzhou, China; b Department of Neonatology, Guangzhou Wowen and Children’s Medical Center, Guangzhou Medical University, Guangzhou, China.

**Keywords:** chondrodysplasia punctata, EBP, gene, mutation, X-linked

## Abstract

**Rationale:**

Chondrodysplasia punctata is a rare hereditary disorder that affects bone development. The disease is primarily caused by mutations in the emopamil binding protein (EBP) gene, leading to X-linked dominant metabolic enzyme abnormalities. This study aims to report a novel EBP mutation in X-linked dominant chondrodysplasia punctata type 2 (CDPX2), expanding the genetic spectrum of the disease and highlighting the critical role of combined clinical and genetic diagnosis in guiding prenatal counseling and postnatal management.

**Patient concerns:**

Multiple prenatal ultrasounds revealed misalignment of the spinal column and short femur. Postnatally, the infant exhibited craniofacial defects, short limbs, congenital ichthyosis, and alopecia.

**Diagnoses:**

Radiographic examination revealed multiple punctate calcifications bilaterally in the pyramids, ischium, pubis, and calcaneus. Whole-exome sequencing of the family revealed a heterozygous mutation, c.204G>A (p.Trp68Ter), in the *EBP* gene in the affected infant, which is a new pathogenic mutation.

**Interventions:**

The infant received continuous positive airway pressure support for respiratory distress, which was discontinued after 7 days due to clinical improvement.

**Outcomes:**

At discharge, respiratory status was stable. Follow-up at 3 months showed significant growth delay: weight 4.6 kg (<3rd percentile) and length 54.5 cm (9th percentile).

**Lessons:**

This case underscores that meticulous physical examination combined with genetic analysis is critical for diagnosing CDPX2. Early identification of *EBP* mutations enables accurate prenatal counseling and risk assessment for future pregnancies.

## 1. Introduction

Chondrodysplasia punctata (CDP) is a rare hereditary disorder that affects bone development. It was first reported in 1914. Its incidence is approximately 1 in 10,000 live births.^[1]^ CDP is characterized by abnormal deposition of calcification in the skeletal cartilage, resulting in a range of developmental abnormalities in both bones and soft tissues.^[2]^ The main cause of CDP is genetic mutations that lead to chromosomal or congenital abnormalities in enzyme metabolism. Based on genetic classification, they can be divided into 5 types: limb root, non-limb root, CDPX1, CDPX2, and Sheffield types.^[3]^

In this article, we present a novel pathogenic emopamil binding protein (EBP) mutation (c.204G > A, p.Trp68Ter) that caused a female case of CDPX2. The clinical phenotype of this infant includes craniofacial defects, short limbs, congenital ichthyosis, and alopecia.

## 2. Case report

The infant was a female with a gestational age of 38 weeks and 2 days, and a birth weight of 3025 g. She had a birth length of 48 cm and a head circumference of 33 cm. Vaginal delivery occurred without asphyxia, and the Apgar scores at 1, 5, and 10 minutes were 10. Fetal ultrasonography revealed misalignment of the spinal column and short femur. Noninvasive DNA tests did not detect any abnormalities. The infant was second-born, and the first-born infant was healthy. Both parents were in good health and there were no congenital defects in their family history. Ethical approval was obtained from the Ethics Committee of Guangzhou Wowen and Children’s Medical Center, Liuzhou Hospital (No. 2025009) and written informed consent was obtained from the patient for publication of this case report details.

**Figure 1.**
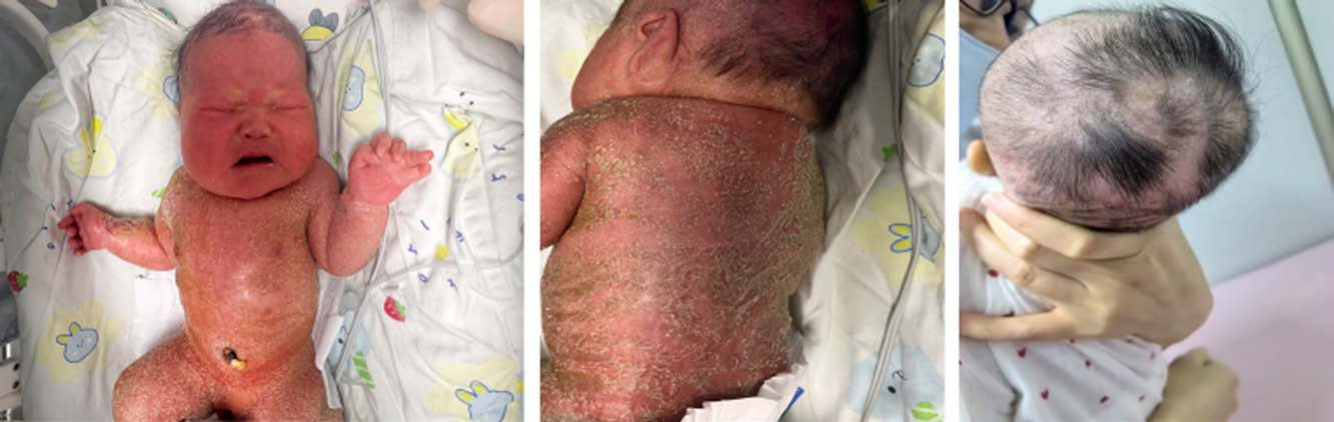
Collapsed nasal bridge, protruding forehead, flat facial profile, congenital ichthyosis, and alopecia.

After birth, physical examination showed a collapsed nasal bridge, protruding forehead, flat facial profile, congenital ichthyosis and alopecia. It is worth noting that there is no syndactyly of the fourth and fifth fingers of the proband’s left hand. The initial appearance may have been due to the angle of the image (Fig. 1). Respiratory assessment indicated shortness of breath with inspiratory retraction, requiring continuos positive airway pressure support. Thoracic deformities were not observed. Limb examination revealed short limbs with slightly increased muscle tension.

**Figure 2.**
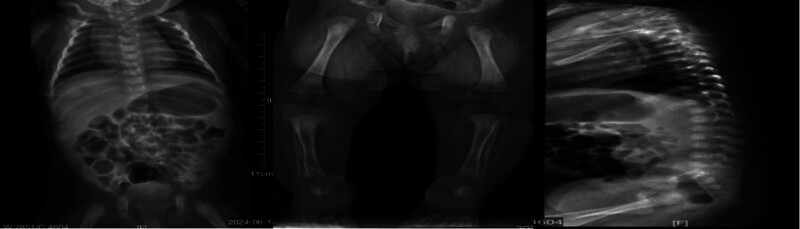
Multiple punctate calcifications bilaterally in the pyramids, ischium, pubis, and calcaneus.

**Figure 3.**
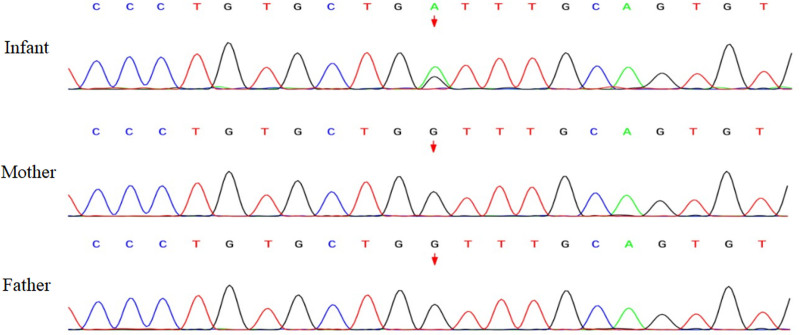
Heterozygous variant in the EBP gene: c.204G>A (p.Trp68 Ter). EBP = emopamil binding protein.

The results of the blood tests, organ ultrasounds, fundus examinations, and hearing screenings were within normal ranges. Echocardiography revealed a patent ductus arteriosus and patent foramen ovale. X-ray imaging revealed multiple punctate calcifications bilaterally in the pyramids, ischium, pubis, and calcaneus (Fig. 2). Whole-exome sequencing analysis detected a heterozygous variant in the EBP gene: NM_006579.3: c.204 G > A (p.Trp68 Ter), indicating a substitution from guanine to adenine at nucleotide position 204, resulting in a premature termination codon at amino acid position 68 (p.Trp68 Ter). This mutation is classified as a point mutation with zero allele frequency in reference population databases, and has not been reported by ClinVar database. Neither parent carried this mutation. According to ACMG guidelines, this variant is considered pathogenic (Fig. 3).

During hospitalization, the infant received continuos positive airway pressure support and used moisturizers and keratolytic agents for skin protection. After 7 days in the hospital, noninvasive ventilation was discontinued, resulting in discharge 19 days post-admission. At 3 months of age, the infant exhibited weight (4.6 kg) below or at the P3 percentile and had a length of 54.5 cm (P9), indicating a notable growth delay.

## 3. Discussion

The CDPX2 (OMIM: 302960) is classified as an X-linked dominant disorder resulting from deletion mutations in the EBP gene, located on the short arm of chromosome X (Xp11). To date, nearly 100 mutation sites have been identified.^[4]^ Herman et al^[5]^ reported that, among 26 suspected female cases of CDPX2, 22 exhibited EBP gene mutations, including 13 novel variants. The EBP gene resides on the short arm of the X chromosome, and while most affected individuals are female, male carriers have been reported. However, CDPX2 is typically lethal in males. The clinical manifestations of CDP are diverse and can affect multiple systems, including alopecia, ichthyosis, flat facial features, shortened limbs, polydactyly, joint contractures or dislocations, tracheal stenosis, cataracts, congenital heart disease, and more.^[6–8]^ A characteristic radiographic finding in individuals with CDP is the presence of abnormal bone calcification in the long bones, vertebrae, and scapulae. It is characterized by uneven bone density and spotty calcification observed in the epiphysis or joint soft tissue area.^[9]^ In this case, the infant presented with typical clinical and imaging findings at birth, accompanied by a de novo pathogenic mutation in the EBP locus, with the clinical phenotype being consistent with the genome. Furthermore, Previous studies have demonstrated that the majority of infants diagnosed with CDP exhibit impaired growth and experience delays in both motor and cognitive development. Impaired growth was also observed during the post-discharge follow-up period in this case.

Although the c.204G > A (p.Trp68Ter) mutation in our case is not listed in public databases, the c.203G > A (p.Trp68Ter) mutation has been reported by Has et al.^[10]^ We acknowledge that our case shares similarities with the CHH syndrome (CDPX2) cases described in the literature, particularly regarding the skin symptoms and gender. However, we also recognize distinct differences in phenotype, such as the absence of cardiovascular abnormalities, variations in skeletal manifestations, and the lack of clear evidence for anticipation (the stepwise increase in disease expression across generations). These differences suggest a complex genotype–phenotype relationship, which underscores the need for further investigation.

The diagnosis of CDPX2 requires differentiation from other conditions including lethal chondrodysplasia, osteogenesis imperfecta type II, and chondrodysplasia punctata. These conditions also present with short-limb malformations; however, they lack spotty calcification at the epiphysis and do not exhibit accompanying mutations in the EBP gene among affected infants. Furthermore, there is currently no specific therapeutic intervention for the treatment of CDPX2. Despite a generally favorable prognosis in most infants, they commonly exhibit diminished stature during adulthood.

This study has several limitations. First, it is a single-case report, limiting generalizability. Second, functional analysis of the c.204G > A variant was not performed to confirm its pathogenic mechanism. Third, long-term follow-up data are needed to assess developmental outcomes.

To summarize, owing to the relatively low incidence of CDP and its phenotypic variability ranging from mild to severe, careful physical examination and genetic testing play a crucial role in the diagnosis of CDPX2. For suspected cases of fetuses exhibiting short bones during prenatal ultrasound screening, genetic testing should be performed to confirm diagnosis. This approach not only aids in assessing the potential risk associated with the fetus, but also enables more precise guidance and advice for expectant mothers.

## Author contributions

**Data curation:** Yanxing Lv.

**Investigation:** Pianpian Pan.

**Resources:** Yongjiang Jiang.

**Writing – original draft:** Xiaoping Shi.

**Writing – review & editing:** Yueju Cai.
